# Latent variable modeling and its implications for institutional review board review: variables that delay the reviewing process

**DOI:** 10.1186/s12910-015-0050-8

**Published:** 2015-08-27

**Authors:** Dong-Sheng Tzeng, Yi-Chang Wu, Jane-Yi Hsu

**Affiliations:** Kaohsiung Armed Forces General Hospital, No. 2, Chung-Cheng 1st Road, Kaohsiung City, Taiwan; Tri-Service General Hospital Beitou Branch, Taipei City, Taiwan; Institute of Aviation and Space Medicine, National Defense Medical Center, Taipei, Taiwan; Department of Surgery, Kaohsiung Armed Forces General Hospital, Kaohsiung City, Taiwan

## Abstract

**Background:**

To investigate the factors related to approval after review by an Institutional Review Board (IRB), the structure equation model was used to analyze the latent variables ‘investigators’, ‘vulnerability’ and ‘review process’ for 221 proposals submitted to our IRB.

**Methods:**

The vulnerability factor included vulnerable cases, and studies that involved drug tests and genetic analyses. The principal investigator (PI) factor included the license level of the PI and whether they belonged to our institution. The review factor included administration time, total review time, and revision frequency. The revision frequency and total review time influenced the efficiency of review.

**Results:**

The latent variable of reviewing was the most important factor mediating the PIs and vulnerability to IRB review approval. The local PIs moderated with genetic study and revision frequency had an impact on the review process and mediated non-approval.

**Conclusions:**

Better guidance of the investigators and reviewers might improve the efficiency with which IRBs function.

## Background

Institutional Review Boards (IRBs) assess research proposals to ensure that they adhere to research regulations, adequately protect the rights and welfare of study participants, and are ethically sound. Accreditation of an IRB by the National Health Institute ensures that human safety is not compromised during the conduct of approved medical research. An IRB should assess the adequacy of structural, procedural, and performance-related aspects of all studies brought before it. Performance problems related to the absence of systematic assessment of the outcomes of this system of oversight have not been addressed [1]. There is no mechanism at the national level to gather systematic evidence on the intersection between research and IRB review [2].

Many investigators have expressed dissatisfaction with the IRB system, characterizing it as being dysfunctional [3], overburdened [2, 4], and over-reaching [5]. Principal investigators (PIs) complain that the problems of the review process conducted by IRBs include the inefficiency with which it identifies irrational decisions [6] and its inconsistency [7]. Research sponsors object that IRB review is time consuming, and that the associated delays can significantly increase the costs of research. The current IRB system has also been described as outdated and inappropriate for the scope and type of research being conducted in the 21st century [8]. Proposals to improve the performance of IRBs include increasing the efficiency of the review process and reducing the amount of time devoted to administrative tasks prior to board review [1].

The significant demands on the time and resources of PIs that are associated with addressing the various challenges associated with IRB approval reduce the willingness of PIs to participate in future research projects [9]. The greatest delay in approval is seen early in the IRB process, with PIs requiring an average of 45.1 ± 31.8 days to submit their study to the IRB [10]. The U.S. National Institutes of Health has suggested training programs for junior PIs and research staff to educate them more about the importance of the protection of human research subjects and the IRB review process [11]. However, there are no reports of the extent to which better appreciation by PIs of patient rights and the IRB process affects the likelihood of a positive outcome of the IRB process.

With regard to vulnerability, vulnerable groups are commonly understood to include pregnant women, prisoners, children, and incompetent adults. Who is a research object? [12] have highlighted the challenges associated with trying to protect such vulnerable groups during studies that involve new drugs. Reporting of serious adverse drug events to IRB helps to promote the safe use of pharmaceuticals [13]. A study by [14] indicated that descriptions of adverse events associated with 15 drugs and reported in clinical practice as structured event descriptions were 2- to 10-fold more complete than the descriptions of the same events in the FDA safety databases. Another important issue relates to obtaining informed consent from vulnerable groups in studies that involve genetic research; members of these groups can pose challenging questions related to ethical and regulatory standards. The complexity and controversial nature of many ethical issues in genome research reflects the limited common ground between genetic researchers and IRB professionals [15] and differences between these two communities in disclosing contradictory research findings [16]. However, we do not know how these variables affect vulnerable groups and the likelihood of IRB approval.

Despite recognition of the need to evaluate the effectiveness of IRB review, no published study has evaluated the effectiveness of the complicated IRB structural model. Several studies that evaluated the structure, process and outcome of IRB review have documented inconsistencies and inefficiencies [7]. Reports on the most important issues concerned with IRB approval have focused on three areas: (1) the total time of review and the rejection frequency; (2) the license level and institution of the PI; and (3) the use of vulnerable groups in drug and genetic studies. Little is known about the complicated relationships between PIs, IRBs, and vulnerable groups. To investigate the factors involved in IRB review and approval, we analyzed research reviewed by our IRB over a 5-year period, using the structure equation model (SEM) for assessment of the latent variables of IRB review, PIs, and vulnerable groups.

## Methods

### Study design

In December 2012, we administered a retrospective survey of the proposals submitted for review by our IRB between January 2008 and December 2012. The study design met the requirements of the Helsinki Declaration and the design was approved by the IRB of the Armed Forces Kaohsiung General Hospital. The proposals were decoded for the names of the PIs and reviewers. We established whether the researchers belonged to our organization and collected details regarding their license level. The four license levels included medical ethics credit from the organization for 6 h per year, >6 h per year, credit from the Department of Health, and credit from a collaborative institutional training initiative (CITI) program qualification. We defined the vulnerability of the population (vulnerable/non-vulnerable), type of risk (drug/non-drug), and need to protect the confidentiality of the information collected (genome/non-genome). The review included: (1) administration time required by the Secretary of the IRB to check on informed consent and proposal documents; (2) total review time for informed consent, comparison of risks and benefits, and recruitment of the test population by scientific and non-scientific reviewers; and (3) the frequencies of revisions and resubmissions by the PIs after the primary and secondary reviews.

### Study data

We surveyed 221 proposals, including human studies, which were submitted to our IRB over a 5-year period. Proposals for which the application was withdrawn before completion of the review were excluded. The secretary of our IRB checked the documents against the checklist. The documents included details related to the study method and materials, the recruitment plan, levels of risk and benefit in the study population, inclusion of vulnerable cases (our definition of vulnerable cases included children, pregnant women, patients with mental illness, prisoners, students, and patients with disability), payment and compensation plan, informed consent, the license held by the PIs and their assistants, whether the PIs worked in our organization, protection of human subjects, and privacy and data confidentiality. If the proposal involved a genetic study, the PIs were required to have a qualification in the ethics of genetic research by Human Research Act of Taiwan. We asked for a full-board review for drug studies, such as phase-one through to phase-four clinical trials. The clinical trials were assigned to full board review. If these documents were complete, the proposal was sent to the Chair of the IRB within 3 days. If not, it was returned to the PIs. The revision frequency was recorded to assess the performance of the PIs.

Our IRB comprises 15 members: six non-scientific reviewers, nine scientific reviewers, six women, nine men, six members who were not affiliated with our organization, and nine members employed by our organization. All IRB members receive annual accreditation in research ethics and undertake this work on a voluntary basis.

Before discussion by the IRB, one non-scientific and one scientific reviewer were assigned by the Chair of the IRB to review the proposal. The total review time was limited to 2 weeks and the duration of the review was recorded for further analysis. There were four outcomes of the review: ‘agree’, ‘agree with revision’, ‘review again after revision’, and ‘not recommended’. These outcomes were forwarded to the full board for further discussion every month. We classified the board review outcome as approval or not; whereas ‘agree’ and ‘agree with revision’ were assigned to the approval group, the other two outcomes were assigned to the control group.

### Statistical analysis

We used the SPSS version 20 statistical software package with the analysis of moment structure (AMOS). The SEM was applied to test the mediating variables between the review outcome and the observed variables. The SEM clarified the extent of the relationships between the variables as well as the chain of cause and effect. In other words, SEM results did not merely show the empirical relationships between the variables when defining the practical situation. For this reason, SEM was used to test the hypotheses. This study also used several indices to evaluate overall model fitness, including the *χ*^2^ test of absolute fit (*p* > 0.05), test of relative fit by the goodness-of-fit index (GFI > 0.90), the adjusted goodness-of-fit index (AGFI > 0.90), the Tucker–Lewis index (TLI > 0.95), the comparative fit index (CFI > 0.90), and the root mean square error of approximation (RMSEA < 0.05). The modeling of latent variables was designed to consider three categories: PIs, review process by reviewers, and the vulnerability of subjects or assessment of risks associated with drug-related and genetic tests. The latent variable of the PI factor included observed variables, such as the license level of the investigators and whether they belonged to our institution. The latent variable of the reviewing process included observed variables, such as administration and secretarial time, board-member review time, and revision frequency. The latent variable of vulnerability included observed variables, such as the involvement of vulnerable cases, and drug and genetic studies. The descriptive analysis involved comparison between the approval group and control group using a cross table and Student’s *t*-test.

## Results

We reviewed 221 proposals submitted to our IRB over a 5-year period. Of these, 159 proposals were approved, 56 were not approved, and six were exempt from a decision. The six exempt reviews were excluded. The type of review (expedited/full board), involvement of vulnerable subjects, involvement of drug use, involvement of genetic analysis, and the duration of PI training did not differ significantly between the group of applications that were approved and those that failed to receive approval. There were significantly more non-approvals for applications from PIs within our organization than for applications from PIs from outside our institution (*p* = 0.014). The administration time required for checking by the secretary of our IRB was longer in the group of applications that were not approved (*p* = 0.008) than in the group that received approval. The revision frequency in the group of applications that were not approved was significantly higher than in the group of applications that were approved (*p* < 0.001). The total review time was significantly longer in the group of applications that were not approved than in the group of applications that were approved (*p* = 0.002) (Table 1).

**Table 1:** Demographic data of the proposals reviewed within the past 5 years by our IRB

	Non-approval	Approval	*X* ^2^∕t	P
	n = 56	n = 159		
Type				
Expedited	50 (89.3 %)	125 (78.6 %)	3.113	0.109
Full-board	6 (10.7 %)	34 (21.4 %)		
Vulnerable case	18 (32.1 %)	59 (37.1 %)	1.154	0.338
Drug study	8 (14.3 %)	13 (8.2 %)	1.754	0.196
Genetic study	16 (28.6 %)	38 (23.9 %)	0.481	0.480
PI training			3.996	0.262
6 h	0	1 (0.63 %)		
> 6 h	2 (3.6 %)	13 (8.2 %)		
DOH	50 (89.3 %)	141 (88.7 %)		
CITI program	4 (7.1 %)	4 (2.5 %)		
Within organization	49 (87.5 %)	108 (67.9 %)	8.565	0.014
Administration time (day)	1. 27 ± 1.51	0. 78 ± 1.03	2.676	0.008
Revision frequency	1. 82 ± 0.57	1. 40 ± 0.76	3.744	0.000
Total review time (day)	47. 8 ± 50.5	31. 4 ± 24.1	3.203	0.002

N = 221, excluded 6 exempt review

*DOH* credits from Department of Health, *CITI* collaborative institutional training initiative

**Fig. 1:**
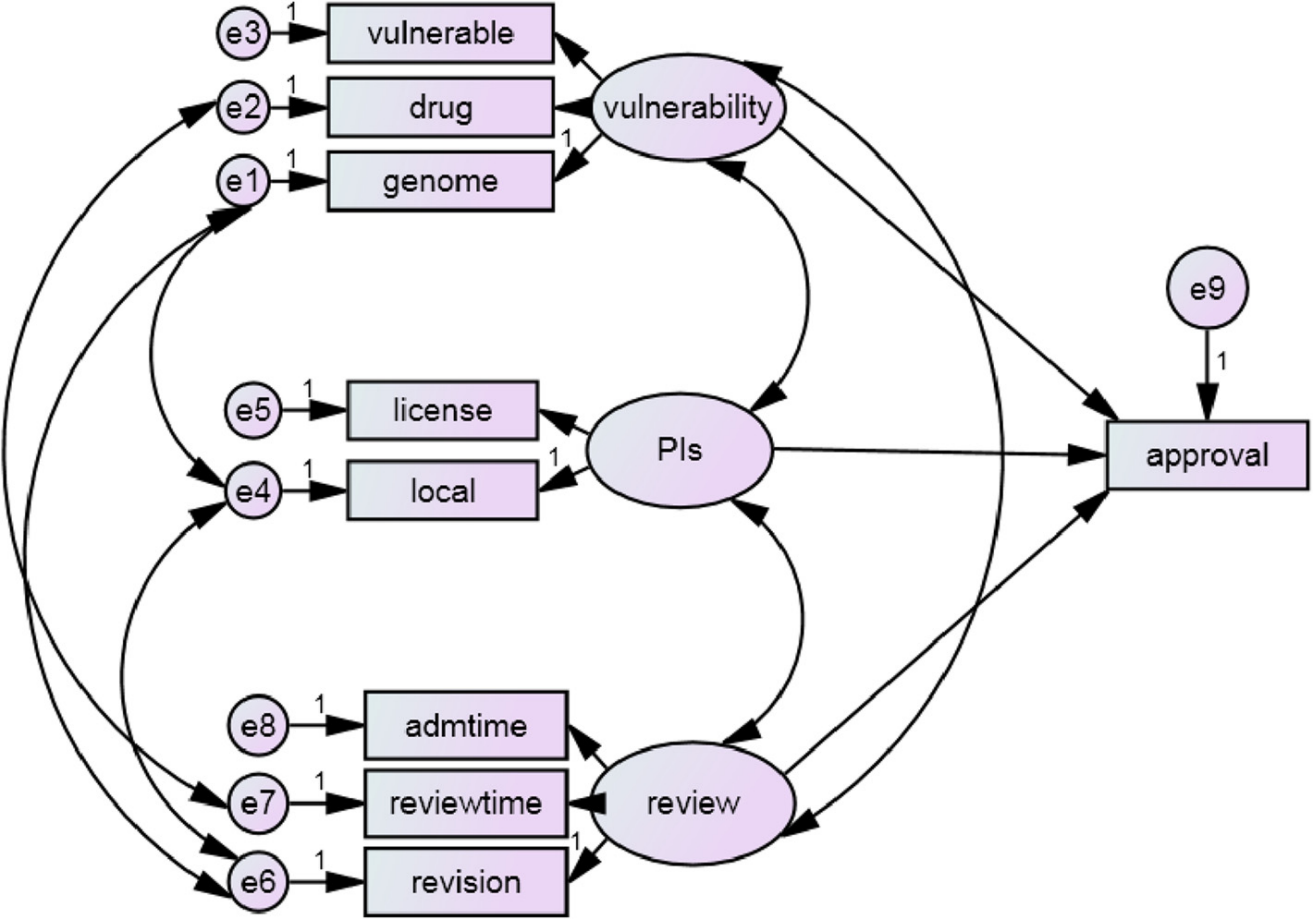
Conceptual construct of the structural equation models of vulnerability, PIs, and review process on the approval outcome

The non-standardized structure equation revealed the interactions between the latent variable of vulnerability and PIs and review (*χ*^2^ = 25.706, degrees of freedom = 18, *p* = 0.107); between the duration of drug study and the duration of review; between the involvement of a genetic study and the revision frequency; between the involvement of a genome study and whether the PIs belonged to our organization; and between the revision frequency and whether the PIs belonged to our organization (Fig. 1).

**Fig. 2:**
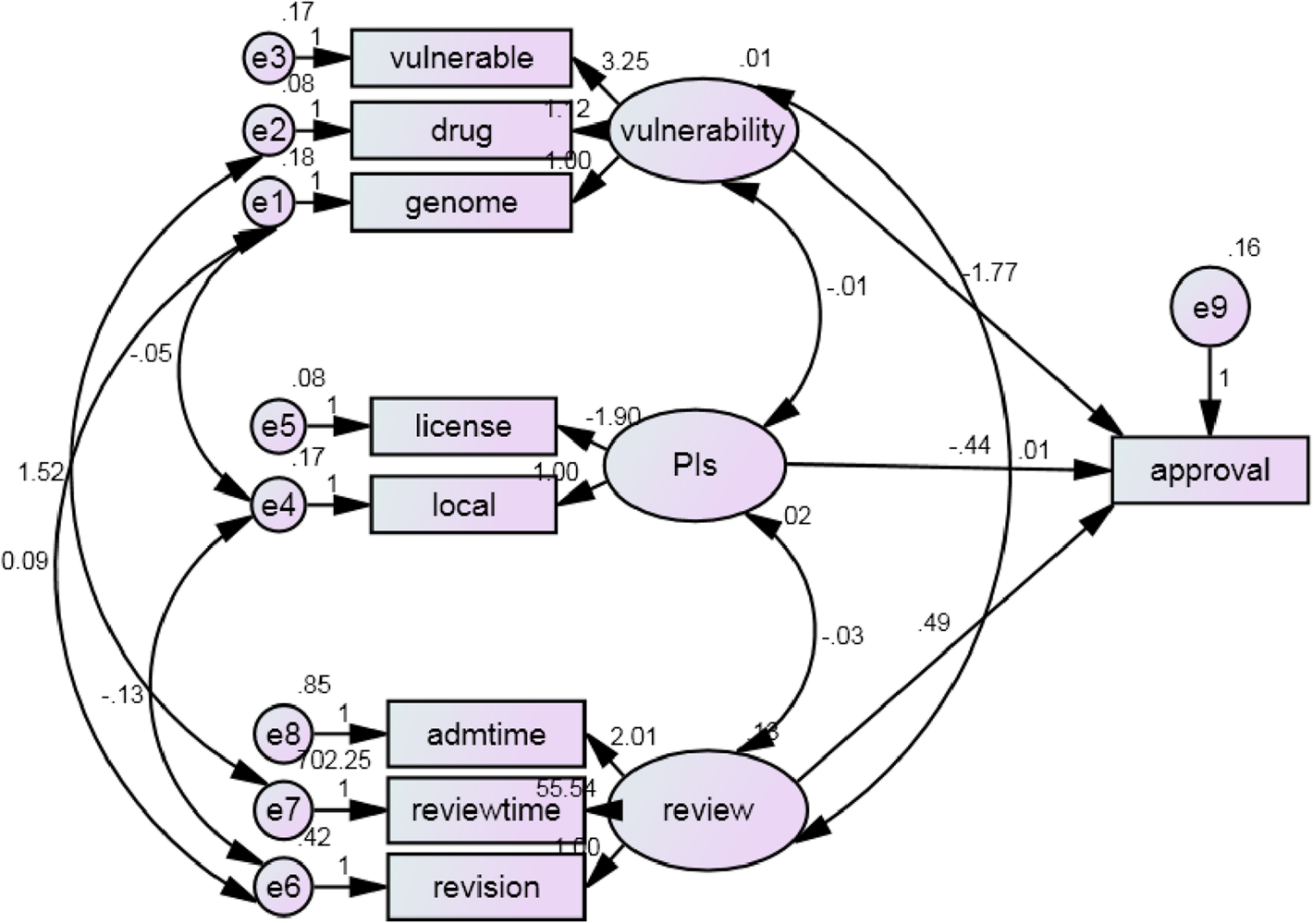
Maximal likelihood estimates of the following model parameters for regression weights, covariance and variance and the theoretical framework on structural models between vulnerability, PIs, and review process on the approval outcome

When estimates covariance among exogenous variables (Fig. 2 and Table 2) revealed the significant relationship between PIs and review time (estimate = −0.031, *p* = 0.04), drug study and review time (estimate = 1.515, *p* = 0.014), genome study and revision frequency (estimate = 0.093, *p* < 0.0001); genome study and whether the PIs belonged to our organization (estimate = −0.047, *p* < 0.0001); and revision frequency and whether the PIs belonged to our organization (estimate = −0.126, *p* = 0.04). When estimates of regression weight, the drug study and vulnerable case revealed no significant (estimate 1.12, *p* = 0.127; estimate 3.25, *p* = 0.117 respectively) to latent variable of vulnerability in genome study set to zero. The license is significant to PI (estimate −1.90, *p* = 0.011) when set the local variable to zero. The total review time and administers time is significant to review (estimate 55.54, *p* < 0.001; estimate 2.01, *p* < 0.001 respectively) when set revision to zero. To approval, review (estimate 0.49, *p* = 0.011) is significant than PI and vulnerability (estimate −0.44, *p* = 0.379; estimate −1.77, *p* = 0.244 respectively). When estimate of variances of exogenous variables, the e1, e2, e3, e4, e5, e6, e7, e8 and d1 (estimate 0.18, 0.08, 0.17, 0.18, 0.08, 0.42, 702.25, 0.85, 0.16; *p* < 0.001, <0.001, <0.001, <0.001, =0.020, <0.001, <0.001, <0.001, <0.001 respectively).

**Table 2:** Estimates of observed and latent variables in the measurement and structural model

	Estimates	S.E.	C.R.	P
PIs ↔ Review	−0.031	0.015	−2.054	0.040
Vulnerability ↔ Review	0.014	0.010	1.399	0.162
Vulnerability ↔ PIs	−0.006	0.005	−1.273	0.203
Genome ↔ Revision frequency	0.093	0.021	4.428	***
Drug ↔ Review time	1.515	0.616	2.460	0.014
Genome ↔ Local PIs	−0.047	0.013	−3.666	***
Local PIs ↔ Revision frequency	−0.126	0.022	−5.645	***

*S.E.* standard error, *C.R.* critical ratios

*** < 0.0001

Confirmation of the model fitness (GFI = 0.975, AGFI = 0.937, TLI = 0.933, CFI = 0.967, and RMSEA = 0.044) revealed that the model was appropriate (Table 3).

**Table 3:** Model identification post modification and test by GFI, AGFI, TLI, CFI, and RMSEA for model fitness

Model	GFI	AGFI	TLI	CFI	RMSEA
Default model	0.975	0.937	0.933	0.967	0.044
Saturated model	1.000			1.000	
Independence model	0.743	0.677	0.000	0.000	0.171

*GFI* goodness-of-fit index, *AGFI* adjusted goodness-of-fit index, *TLI* Tucker-Lewis index, *CFI* comparative fit index, *RMSEA* root mean square error of approximation

## Discussion

This study revealed that revision frequency and total review time influenced reviewing, and that the latent variable of reviewing was the most important factor in mediating the PIs and vulnerability to IRB review approval. And this study is the first application of the AMOS to test the systemic pathway for the hypothesis on the relative delay variables of the reviewing process. AMOS revealed the reviewing process to be the mediator, which is not the only role with the responsibility of non-approval. The latent variables ‘vulnerability’ and ‘PIs’ played a significant role in approval. Many authors have expressed dissatisfaction with the IRB system [2–6]. This is the first use of SEM to provide a more comprehensive assessment of the factors that influence the success of applications brought before IRBs. The SEM found that both the IRB review process and PI factors affected the likelihood of approval of a request. Most of the literature related to the value of IRBs has focused on issues related to their structure and operational processes instead of the measurement of outcomes [1, 9, 10]. In this study, our use of dichotomy approval and non-approval to reflect the effectiveness and efficiency of the IRB meant that other functions of the IRB were ignored. The functions of the IRB include the protection of human subjects and the education of participants in the research. The approval process might be of interest to PIs. Revisions and recommendations from the reviewers need the compliance of the PIs. For the purpose of safety, [1] have outlined an accreditation process and a standardized system for collecting and disseminating data on adverse events and the performance assessment of IRBs. Appropriate arrangements for prospective independent scientific and ethical review are required, as are measures to establish whether the IRB is satisfying federal regulations for research studies that involve human research subjects [7].

Local IRBs like ours, the drug relative study was directly measured as the observed variable for the latent variable of vulnerability. The reason for patient safety is that adverse drug events are monitored regularly [13]. Most common serious adverse events in a clinical setting are related to drugs that are administered in our general hospital. The risk level for consideration of institutional responsibility about drug studies is higher than minimal injury [12]. Indeed, in this study, we found the total review time to be associated with the nature of the drug study. As the path analysis indicated, the total review time acted as a mediator between drug studies and approval. For genome studies, vulnerability was regarded as a latent variable because of the large number of genetic studies conducted worldwide in recent years, even in developing countries. There are no consistent standards in IRB review concerning new technology, specimen storage, disclosure of individual research results, and ethical problems [16]. In the present study, SEM found that PIs in our organization who were involved in genetic studies submitted a larger number of revised research proposals than people who did not conduct genetic research and who conducted genetic research but were not affiliated with our organization. One study found that, compared with IRB members, more genetic researchers trusted the confidentiality of coded data, fewer expected harm from re-identification, and fewer considering re-consent necessary in certain scenarios [15]. Another report suggests that IRB members might inflate the vulnerability of research subjects and their inability to make decisions regarding the relative risks and benefits of research [17]. Regardless of ethical, scientific, or regulatory considerations, drug and genetic studies pose particular challenges to the review process. IRB members and reviewers need regular training about issues common to multiple types of studies, as well as those that are unique to certain fields.

With regard to PIs, those within our organization who submitted requests for genetic studies were frequently asked to revise their proposals. In this public hospital, retired physicians follow the rule of official provision. The retired age is 48 to 52 years old. The reason for this might be that more junior PIs and less experienced research teams were not familiar with the routine checklist for their proposal [1] or that the regulations surrounding genetic studies were complex and thus delayed resubmission [10]. It has been suggested that difficulties and delays with the local IRB approval process sometimes result in investigators deciding to abandon research [9, 18]. The function of any IRB is to not only protect human research subjects, but also to educate researchers about the safe conduct of research that involves human subjects [7]. We found the latent variable of PIs moderated the other latent variable of review, and thus affected approval. Many studies have focused on the review process and have demanded reform of the IRB [1, 7]. Multiple revisions are often associated with different interpretations; however, full review by an IRB is recommended even for the initial review [7, 10]. Inefficiency is the most common complaint about the IRB [6, 7]. In our SEM, the total review time and revision frequency played mediating roles in the review process. The latent variables of vulnerability and PIs interacted with review and acted as moderators of approval. Guidance of the PIs and reviewers would improve the function of the IRB.

### Limitations

There were several limitations to the present study. First, we could not guarantee the use of consistent standards amongst every scientific/non-scientific, affiliated/non-affiliated reviewer who reviewed the proposals. The process or the review system provides reviews to the same standard is a source of concern. Second, we did not make reason analysis about the revision frequency which could not clarify the role of PIs before reviewing process. It needs advanced investigation. Third, the local IRB data and conclusions reported here might not be generalized to apply to other IRBs.

## Conclusions

The revision frequency and total review time influenced the review process, and the latent variable of reviewing was the most important factor responsible for mediating the PIs and vulnerability to IRB review approval. The local PIs moderated with genetic study and revision frequency affected the review process and mediated non-approval. The drug study prolonged the total review time and mediated non-approval. The review process is the mediator between vulnerability, PIs and approval. Guidance of the investigators and reviewers involved in genetic and drug studies would improve the function of the IRB.

## Abbreviations

IRB: Institute review board
PI: Principal investigator
SEM: Structure equation model
AMOS: Analysis of moment structure
GFI: Goodness-of-fit index
AGFI: Adjusted goodness-of-fit index
TLI: Tucker–Lewis index
CFI: Comparative fit index
RMSEA: Root mean square error of approximation
